# Detection of new endemic focus of tick-borne encephalitis virus (TBEV), Hampshire/Dorset border, England, September 2019

**DOI:** 10.2807/1560-7917.ES.2019.24.47.1900658

**Published:** 2019-11-21

**Authors:** Maya Holding, Stuart D Dowall, Jolyon M Medlock, Daniel P Carter, Liz McGinley, Mollie Curran-French, Steven T Pullan, John Chamberlain, Kayleigh M Hansford, Matthew Baylis, Richard Vipond, Roger Hewson

**Affiliations:** 1 National Institute for Health Research Health Protection Research Unit in Emerging and Zoonotic Infections, Liverpool, United Kingdom; 2 Virology and Pathogenesis Group, National Infection Service, Public Health England, Porton Down, United Kingdom; 3 Medical Entomology and Zoonoses Ecology, Emergency Response Department, Public Health England, Porton Down, United Kingdom; 4 Genomics, National Infection Service, Public Health England, Porton Down, United Kingdom; 5 Institute of Infection and Global Health, University of Liverpool, Liverpool, United Kingdom

**Keywords:** encephalitis, tick-borne virus, Ixodes ricinus, zoonoses, flavivirus, vector-borne infections

## Abstract

The presence of tick-borne encephalitis virus (TBEV) was detected in a questing tick pool in southern England in September 2019. Hitherto, TBEV had only been detected in a limited area in eastern England. This southern English viral genome sequence is distinct from TBEV-UK, being most similar to TBEV-NL. The new location of TBEV presence highlights that the diagnosis of tick-borne encephalitis should be considered in encephalitic patients in areas of the United Kingdom outside eastern England.

The geographical spread of tick-borne encephalitis virus (TBEV) is expanding in Europe [1]. In the Netherlands, the first human cases of TBE were recorded in 2016 [1]. In the UK, TBEV was detected in ticks removed from deer in the Thetford Forest area of East Anglia in eastern England in May 2019 [2,3]. Here we report findings of further investigations in Hampshire and its bordering areas in southern England.

## Detection of tick-borne encephalitis virus using deer as sentinels

TBEV is a member of the flavivirus family, causing tick-borne encephalitis (TBE), a neurologic encephalitic disease of humans. Five subtypes of TBEV are known: European (TBEV-Eu), Far Eastern (TBEV-FE), Siberian (TBEV-Sib), Baikalian (TBEV-Blk) and Himalayan (TBEV-Him) [4]. *Ixodes ricinus* is the main tick vector of TBEV-Eu, the predominant subtype in western Europe [5]. Louping ill virus (LIV), vectored by the same tick species, is a member of the TBEV serocomplex that is endemic in areas of the UK where it causes disease in sheep, and on rare occasions, also in humans [6]. The close genetic homology between LIV and TBEV results in cross-reactivity in standard serological assays, therefore the detection of viral nucleic acid is necessary to differentiate between the two viruses.

**Figure 1:**
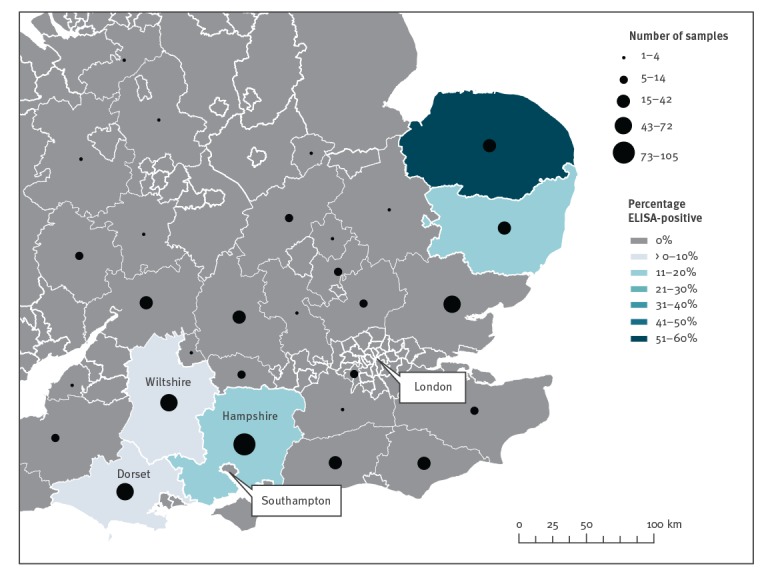
Number of deer samples tested for exposure to tick-borne encephalitis virus serocomplex^a^ and relative percentage of positives, eastern, southern and central England, February 2018–January 2019

Between February 2018 and January 2019, 1,309 deer serum samples were collected from culled deer in England and Scotland as part of a research study; 4% of samples were ELISA-positive for the TBEV serocomplex [2]. Our seroprevalence data highlighted two key geographic areas of interest (Figure 1) that showed evidence of flavivirus seropositivity in deer. Notably, these areas, Thetford Forest on the Norfolk/Suffolk border in eastern England and Hampshire in southern England, have not reported LIV in livestock [7,8]. This raised suspicion that another flavivirus may be present and follow-up investigations were conducted.

TBEV: tick-borne encephalitis virus.

Source: Ordnance Survey and National Statistics data for geographical and administrative boundaries. Adapted from version found in [2].

^a^ Commercial TBEV ELISA was used to determine if samples were positive for antibodies to TBEV serocomplex [2].

## Questing tick sampling

Questing tick surveys were conducted at four sites during July and August 2018 (Table): (i) one on the Hampshire/Dorset border (site 1A) (ii) two in Hampshire (sites 2 and 3), and (iii) one on the Hampshire/Wiltshire border (site 4). The four sites were selected as areas where at least one seropositive deer was previously identified. Additional sampling was conducted on site 1 during June 2019 because this location had the highest concentration of seropositive deer (50%) within Hampshire and its bordering counties in the previous year. Three localities were surveyed at site 1 (1A, 1B and 1C), where 915 ticks were collected and tested during 2018 and 2,155 in 2019.

**Table:** Number of questing ticks tested by site, Hampshire and its borders, England, United Kingdom, 2018 and 2019

Month and year	Site	Area	Nymphs (n)	Adult males (n)	Adult females (n)	Total ticks (n)
July and August 2018	1A	Hampshire/Dorset border	420	25	35	480
	2	Hampshire	160	10	30	200
	3	Hampshire	100	15	20	135
	4	Hampshire/Wiltshire border	90	5	5	100
June 2019	1A	Hampshire/Dorset border	870	100	110	1,080
	1B	Hampshire/Dorset border	430	65	80	575
	1C	Hampshire/Dorset border	340	75	85	500

## Detection of viral RNA

During September 2019, after all tick samples had been collected, ticks were morphologically identified as *Ixodes ricinus* [9] and grouped into pools of 10 nymphs or 5 adult males or 5 adult females. Pooled ticks were homogenised in 300 µl buffer RLT in MK28-R Precellys tissue homogenising tubes using a Precellys 24 homogeniser (Bertin, Montigny-le-Bretonneux, France) [2]. Samples were then passed through a QIAshredder (Qiagen, Hilden, Germany) and extracted using the BioSprint 96 One-For-All Vet Kit (Qiagen) [2]. All tick pools were tested with the LIV/TBEV real-time RT-PCR assay developed by Schwaiger and Cassinotti [10]. RNA was amplified in 20 µL real-time RT-PCR mix containing 0.8 µL Invitrogen SuperScript III with Platinum Taq Mix (ThermoFisher, Waltham, United States), 10 µL Invitrogen 2X Reaction Mix, 1.6 µL of 50 mM MgSO_4_, 1 µL of 1 µM forward primer (F-TBE 1), 1 µL of 18 µM reverse primer (R-TBE 1), 0.2 µL of 25 µM probe (TBE-Probe WT), 5 µL template and 0.4 µL molecular-grade water. One positive pool of a total of 373 pools tested, was detected in an adult female group (Ct 16.12), collected from site 1B on the Hampshire/Dorset border. The minimum infection rate of ticks infected with TBEV in site 1B was estimated as 0.17% [11].

## Genome sequencing and phylogenetic analysis

**Figure 2:**
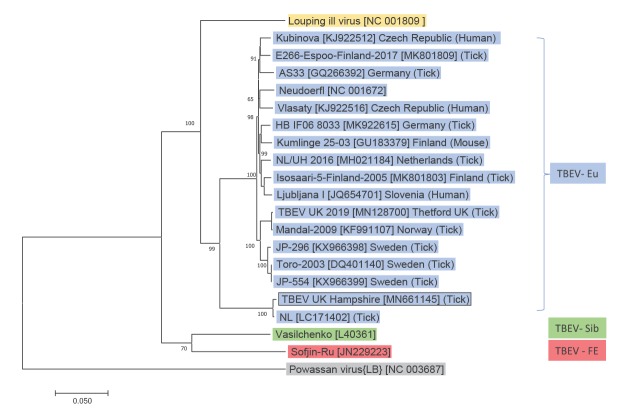
Phylogenetic relationship of contemporary strains of tick-borne encephalitis virus (TBEV) and TBEV-UK Hampshire, England, United Kingdom, 2019

The one pool positive for TBEV RNA was sequenced metagenomically using the Oxford Nanopore GridION [12] and the complete TBEV coding sequence was obtained: TBEV-UK Hampshire, GenBank accession number MN661145. Data was compiled with a range of other published TBEV genomes circulating in Europe, together with reference genomes from other TBEV subtypes to infer the evolutionary history. Figure 2 shows this phylogenetic relationship and indicates that TBEV-UK Hampshire is most closely related to TBEV-NL (LC171402.1), a strain of TBEV detected in ticks in the Netherlands in 2017 [3]. When compared with the TBEV-NL strain, TBEV-UK2 Hampshire contains 49 single nt polymorphisms leading to 12 amino acid substitutions within the coding sequence.

The box highlights the ticks collected in Hampshire in 2019. The tree was constructed with a maximum-likelihood analysis of full length genomes and is rooted with the tick-borne Powassan virus. European TBEV strains are highlighted in blue, Siberian TBEV in green, Far Eastern TBEV in pink, and Louping ill virus in yellow. Strains are identified with the name, GenBank accession numbers, country location and host.

## Discussion and conclusion

Our findings indicate that TBEV prevalence in ticks is not limited to the Thetford Forest area in eastern England, but also includes the Hampshire/Dorset border in southern England. The viral genome sequence obtained from ticks in southern England is most similar to a virus identified in 2017 in the Netherlands [3] and is distinct from the TBEV-UK discovered in the Thetford Forest area in May 2019 [2]. The identification of two distinct TBEV-Eu genomes in the UK provides compelling evidence of two separate importation events into the UK. Birds such as thrushes transport large numbers of ticks over great distances during autumn migration, when many travel to the UK from TBEV-endemic areas in northern and western Europe, including the Netherlands [13,14]. Factoring bird migration routes, the locality of the TBEV-UK Hampshire genome detection in southern England and its close homology to the Netherlands genome suggests that importation of TBEV-UK Hampshire to the UK may have occurred through the transport of infected ticks carried on migratory birds.

Additionally, the presence of TBEV in questing ticks indicates an established enzootic cycle involving ticks and other wildlife hosts, supporting the hypothesis that TBEV is established in the UK and is being maintained in enzootic cycles.

The estimated prevalence of 0.17% in this identified focus is relatively low when compared with some other reports from mainland Europe [15]. As TBEV foci comprise of defined boundaries, a possible explanation could be that the centre of this focus was not detected on this sampling occasion [16]. Follow-up investigations will be conducted to identify the exact location and boundaries of the endemic focus.

The risk of TBEV to the general population in the UK is currently assessed to be very low [17], and there have been no autochthonous confirmed cases of TBE in the UK to date. However, a probable case diagnosed through serology alone has been traced back to a tick bite received at a location in Hampshire close to where the TBEV-positive tick pool was collected [18]. These data reinforce the need to consider TBEV infection as a potential diagnosis in encephalitis patients, particularly those with history of tick bite. However, confirmation of TBE in the UK is complicated by the circulation of LIV, which is cross-reactive in standard serological tests. Further work is required to identify risk areas in the UK where climatic and ecological conditions may support the maintenance of TBEV.
